# Supplementary material for: Astrogliosis Releases Pro-Oncogenic Chitinase 3-Like 1 Causing MAPK Signaling in Glioblastoma
Source: Cancers (Basel). 2019 Sep 26;11(10):1437. doi: 10.3390/cancers11101437 (PMC6826948; doi:10.3390/cancers11101437)
Supplement: Supplementary file 1 [file cancers-11-01437-s001.pdf]

Supplementary materials

Astrogliosis Releases Pro-Oncogenic Chitinase 3-Like 1 Causing MAPK Signaling in Glioblastoma

Julian Wurm, Simon P. Behringer, Vidhya M. Ravi, Kevin Joseph, Nicolas Neidert, Julian P. Maier, Roberto Doria-Medina, Marie Follo, Daniel Delev, Dietmar Pfeifer, Jürgen Beck, Roman Sankowski, Oliver Schnell and Dieter H. Heiland

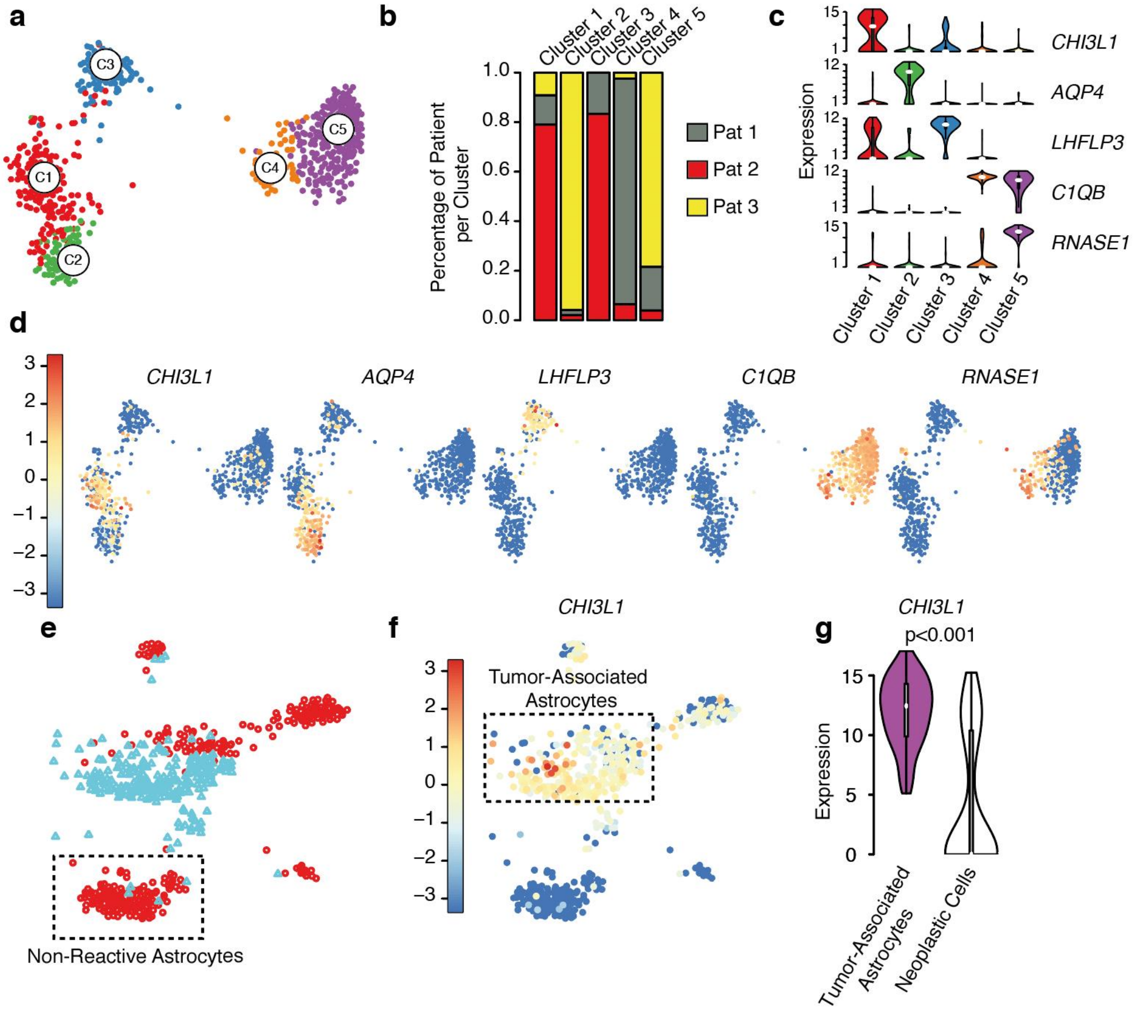

Figure S1. CHI3L1 is presented in tumor-associated astrocytes. (**a**) Extraction of neoplastic cells from the Darmanis data set. RaceID clustering reveals 5 different clusters visualized as a t-sne map. (**b**) Cells from each individual cluster be observed across all patients with high diversity regarding inter-patient distribution. (**c**) CHI3L1 is found be the marker gene in cluster 1. (**d**) T-sne heatmaps of all marker genes. CHI3L1 expression is observed in cluster1 and 2, other neoplastic clusters do not express CHI3L1. (**e,f**) Astrocytes are extracted from the same dataset. After RaceID clustering, reactive astrocytes from the tumor reveal a unique expression signature marked by CHI3L1. Compared to neoplastic cell populations, the astrocytic CHI3L1 expression is more homogeneous distributed in reactive astrocytes. (**g**) The global expression level of CHI3L1 is higher expressed in tumor- associated astrocytes.

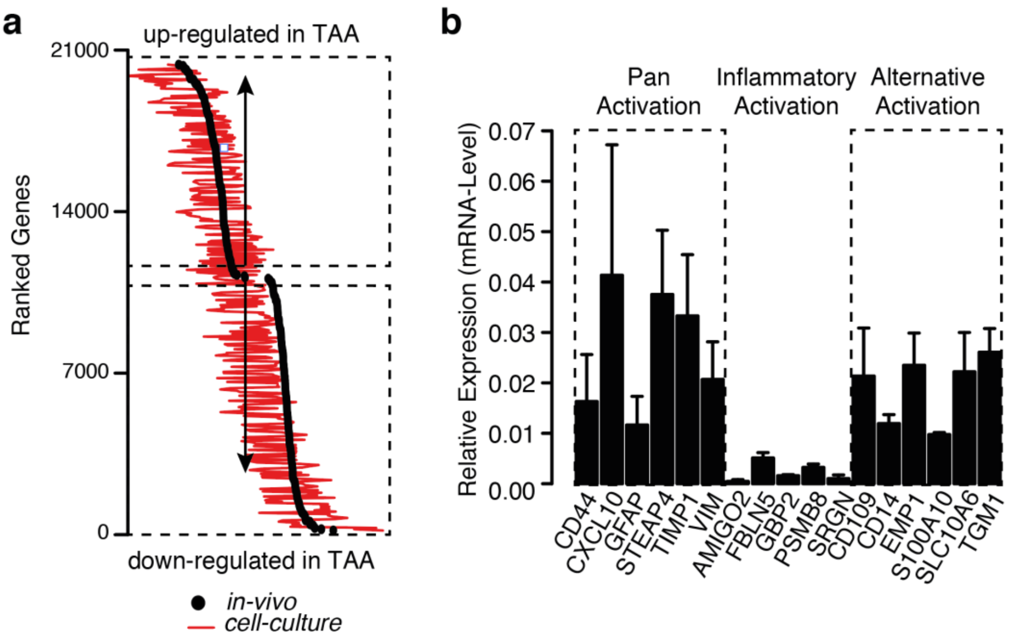

Figure S2. Comparison between cell-model and in-vivo data. (**a**) Visualization of differently expressed genes from purified astrocytes (black) and aligned rank from differently expressed genes from astrocytes cultured naive or in coculture (red). (**b**) RT-qPCR analysis of pan-, inflammatory- and alternative activation based on the signature gene expression (*36*).

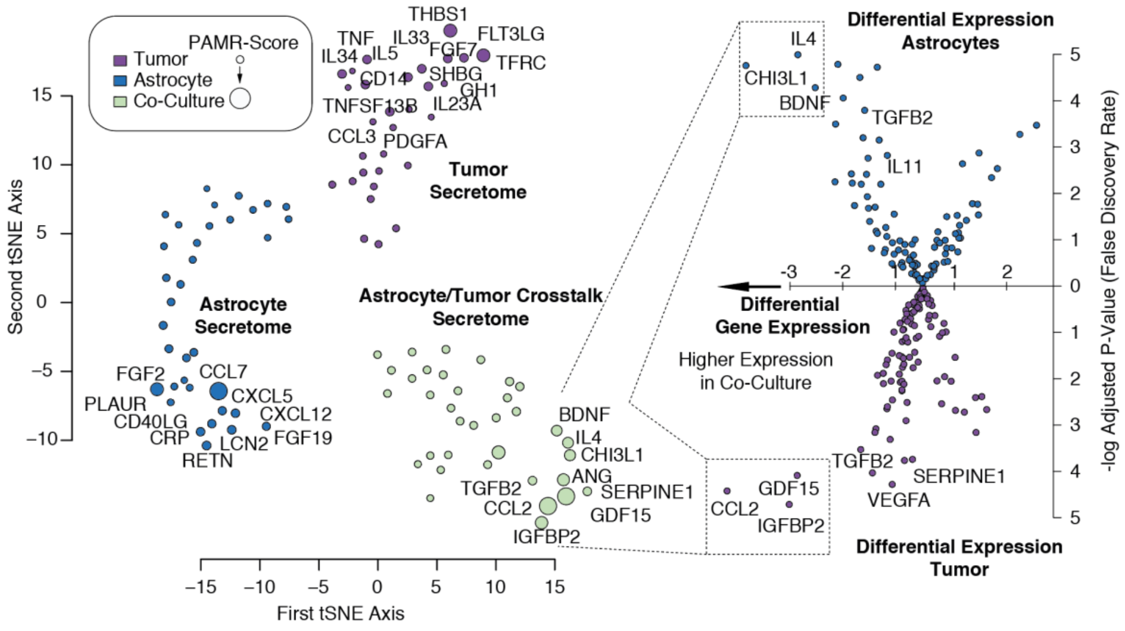

Figure S3. Co-aligned cytokine and expression data. tSNE two-dimensional map of secretome profiling including astrocytes, tumor cells and co-cultures of tumor cells/astrocytes. Colors and size indicate the identity of each condition subclass based on a Predictive Analysis for Microarrays (PAMR) computational model.

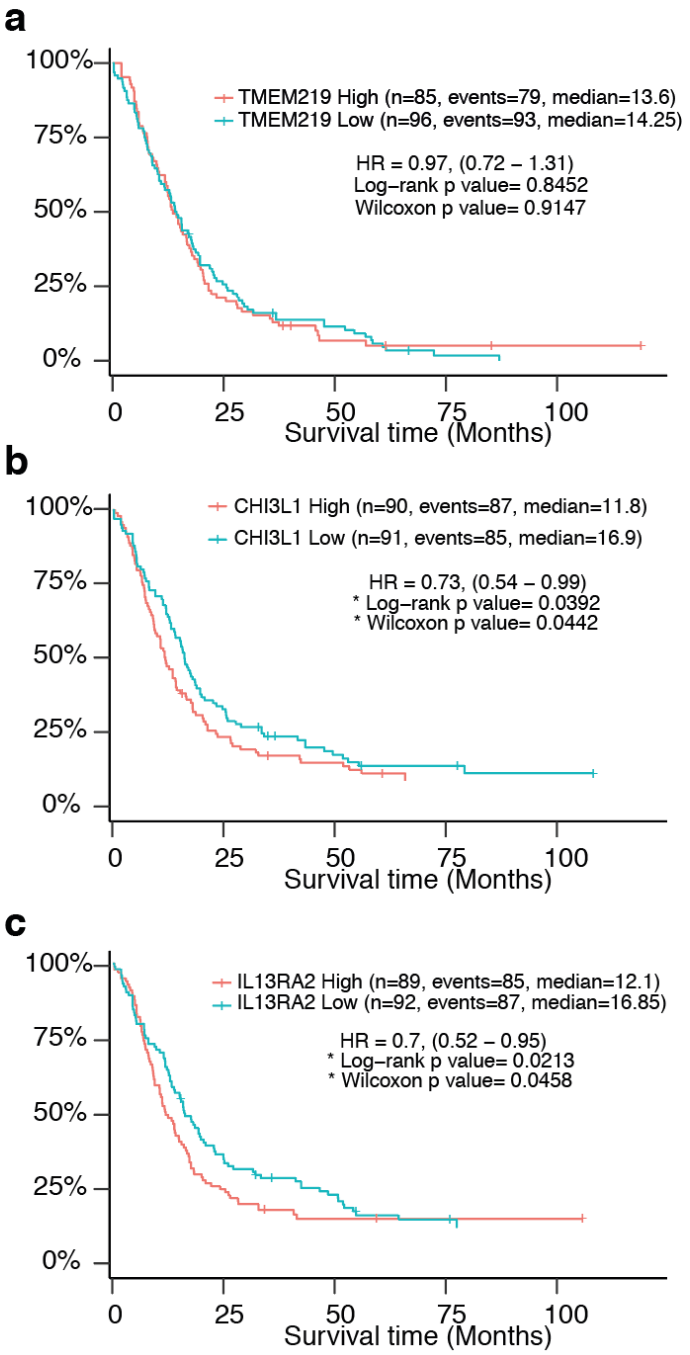

Figure S4. Survival of glioblastoma patients based on gene-expression data. Kaplan-Meier survival statistics based on gene expression levels of TMEM219 (**a**), CHI3L1 (**b**) and IL13RA2 (**c**).

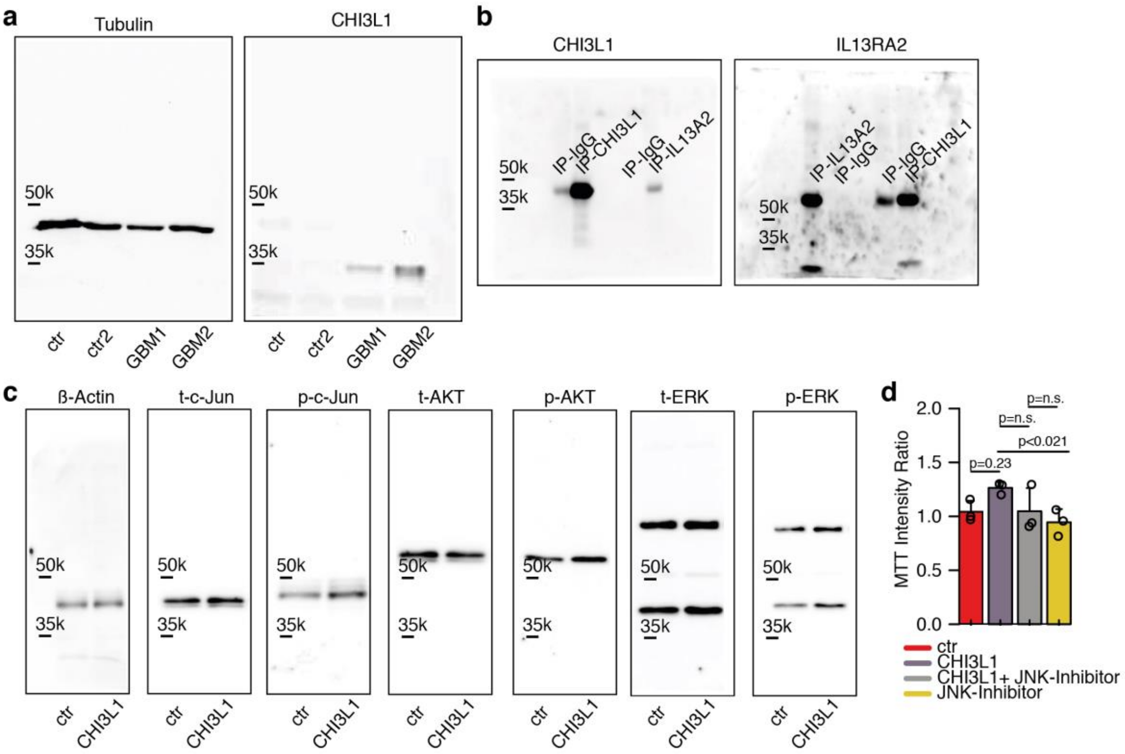

Figure S5. Original western blots and additional data. (**a**) Western blots from Figure 1d (**b**) Western blots from Figure 3i, (**c**) Western blots from Figure 3f. (**d**) MTT assay of CHI3L1 treatment and JNK inhibition after 24 h. Ratios are given as mean ± standard deviation. *p*-values are determined by one-way ANOVA.

© 2019 by the authors. Licensee MDPI, Basel, Switzerland. This article is an open access article distributed under the terms and conditions of the Creative Commons Attribution (CC BY) license (http://creativecommons.org/licenses/by/4.0/).
